# Cardiovascular diseases as risk factors of post-COVID syndrome: a systematic review

**DOI:** 10.1186/s12889-024-19300-4

**Published:** 2024-07-10

**Authors:** Nur Insyirah Sha’ari, Aniza Ismail, Aznida Firzah Abdul Aziz, Leny Suzana Suddin, Amirah Azzeri, Ruhana Sk Abd Razak, Nur Syazana Mad Tahir

**Affiliations:** 1 Department of Public Health Medicine, Faculty of Medicine, Universiti Kebangsaan Malaysia, Cheras Campus, Bandar Tun Razak, Cheras, Kuala Lumpur, 56000 Malaysia; 2 Faculty of Public Health, Universitas Sumatera Utara, North Sumatra, Jalan Universitas No. 21 Kampus USU, Medan, 20155 Indonesia; 3 Department of Family Medicine, Faculty of Medicine, Universiti Kebangsaan Malaysia, Cheras Campus, Bandar Tun Razak, Cheras, Kuala Lumpur, 56000 Malaysia; 4 Department of Public Health Medicine, Faculty of Medicine, Universiti Teknologi MARA, Sungai Buloh Campus, Sungai Buloh, 47000 Selangor Malaysia; 5 Department of Primary Care, Faculty of Medicine and Health Sciences, Universiti Sains Islam Malaysia, Persiaran Ilmu, Putra Nilai, Negeri Sembilan, Nilai, 71800 Malaysia; 6 Federal Government Administrative Centre, Ministry of Health Malaysia, Pusat Pentadbiran Kerajaan Persekutuan, Wilayah Persekutuan Putrajaya, Putrajaya, 62000 Malaysia

**Keywords:** Chronic long COVID-19, Heart diseases, Hypertension, Heart failure, Myocardial infarction, Ischaemia, Heart attack, Peripheral diseases, Cardiac arrhythmia

## Abstract

**Background:**

A growing proportion of people experience incomplete recovery months after contracting coronavirus disease 2019 (COVID-19). These COVID-19 survivors develop a condition known as post-COVID syndrome (PCS), where COVID-19 symptoms persist for > 12 weeks after acute infection. Limited studies have investigated PCS risk factors that notably include pre-existing cardiovascular diseases (CVD), which should be examined considering the most recent PCS data. This review aims to identify CVD as a risk factor for PCS development in COVID-19 survivors.

**Methods:**

Following the Preferred Reporting Items for Systematic Review and Meta-Analyses (PRISMA) checklist, systematic literature searches were performed in the PubMed, Scopus, and Web of Science databases from the earliest date available to June 2023. Data from observational studies in English that described the association between CVD and PCS in adults (≥ 18 years old) were included. A minimum of two authors independently performed the screening, study selection, data extraction, data synthesis, and quality assessment (Newcastle-Ottawa Scale). The protocol of this review was registered under PROSPERO (ID: CRD42023440834).

**Results:**

In total, 594 studies were screened after duplicates and non-original articles had been removed. Of the 11 included studies, CVD including hypertension (six studies), heart failure (three studies), and others (two studies) were significantly associated with PCS development with different factors considered. The included studies were of moderate to high methodological quality.

**Conclusion:**

Our review highlighted that COVID-19 survivors with pre-existing CVD have a significantly greater risk of developing PCS symptomology than survivors without pre-existing CVD. As heart failure, hypertension and other CVD are associated with a higher risk of developing PCS, comprehensive screening and thorough examinations are essential to minimise the impact of PCS and improve patients’ disease progression.

**Supplementary Information:**

The online version contains supplementary material available at 10.1186/s12889-024-19300-4.

## Introduction

Coronavirus disease 2019 (COVID-19) has caused a significant burden worldwide. The clinical spectrum of severe acute respiratory syndrome coronavirus 2 (SARS-CoV-2) infections can range from asymptomatic infection through respiratory disease, to multi-organ failure, and death [1]. The common symptoms include fatigue, dyspnoea, palpitations, sleep difficulties, and anxiety [2]. There are hundreds of millions of COVID-19 survivors worldwide, of whom some reported experiencing incomplete recovery months after contracting the acute illness, which is a condition known as post-COVID syndrome (PCS) [3–5]. While the acute stage of COVID-19 was identified early, the underlying aetiology of persistent and varying symptomatology of PCS remains inadequate [6].

PCS is described as a condition where patients develop several persistent symptoms for > 12 weeks after acute COVID-19 infection and that cannot be explained by any alternative diagnosis [7]. The estimated prevalence of PCS is between 10% and 35% [8, 9]. The long-term effects on physical and mental health constitute a rising public health problem and a serious challenge for healthcare systems. Thus, the critical understanding of the predisposing elements of PCS development should be emphasised to enable the identification of the significant determinants [10]. Clinicians would benefit from being able to quickly provide the correct treatment and support with a whole-patient view to reduce morbidity and improve outcomes. This would be facilitated by being able to identify the groups most at risk for PCS [11].

The documented incidence of PCS varies significantly between and within several nations, including the UK (1.6–71%), Africa (68%), Italy (5–51%), India (22%), China (49–76%), and the USA (16–53%) [12]. When compared to community studies such as Sudre et al. [13], studies that evaluated hospitalised patients typically reported higher prevalence estimates, e.g. 76% in Huang et al. [14] and 71% in Evans et al. [15], which reflects the complex relationship between acute illness severity, comorbidities, and persistent symptoms. The substantial variation in prevalence estimates among the different studies might have been due to variations in the study population such as sociodemographics, clinical practices and treatment protocols, duration of follow-up etc. [4].

A higher risk of developing PCS was associated with a gradient increase in age, female, hospitalisation during acute COVID-19, symptom load including dyspnoea and chest pain, and the existence of comorbidities such as asthma [13, 16]. Galal et al. [17] reported that 26.5% of PCS patients had chronic diseases, and hypertension was the most significant comorbidity associated with PCS, followed by chronic pulmonary diseases. These CVD conditions included pericarditis (hazard ratio [HR] = 1.85, 95% confidence interval [CI]: 1.61–2.13), heart failure (HR = 1.72, 95% CI: 1.65–1.80), ischemic heart disease (HR = 1.72, 95% CI: 1.56–1.90), and atrial fibrillation (HR = 1.71, 95% CI: 1.64–1.79) [18].

Presently, studies that discuss the risk factors associated with PCS, specifically pre-existing comorbidities such as CVD, are limited. It is a critical necessity to understand CVD as a risk factor for PCS in the literature. Furthermore, the effect of medical disorders specifically CVD on PCS patients and whether the spectrum differs from that of patients without CVD must be investigated considering newly available data on PCS. Therefore, this systematic review aimed to identify CVD as the risk factor for PCS development. Thus, it would enable the identification of people who are at risk, assist with early screening and diagnosis, and establish suitable management that better serves their requirements [19, 20].

## Methods

This systematic review aimed to identify the association between CVD and PCS worldwide and was designed and conducted following Preferred Reporting Items for Systematic Review and Meta-Analyses (PRISMA) guidelines [21]. The registration protocol was registered with the International Prospective Register of Systematic Reviews (PROSPERO ID: CRD42023440834). The authors designed the research questions, study protocol, search strategies, and eligibility criteria independently. PCS was defined as a condition when patients experience new or persistent COVID-19 symptoms for > 12 weeks after acute SARS-CoV-2 infection that cannot be explained by any alternative diagnosis.

### Data sources and searches

The relevant search terms were identified using medical subject heading (MeSH) phrases and synonyms correlated to the review topic. A systematic search was performed with expert librarian support using the Scopus, PubMed, and Web of Science (WOS) electronic databases without language restriction. Studies published from 1 January 2020 to June 2023 were included in the search. The search also involved PCS- and CVD-related terms, and the whole search term strategy was constructed with Boolean operators (outlined in Table 1). After retrieving and compiling the identified references from all databases in Endnote version 20.6, duplicates were eliminated, and the shortlisted publications were transferred to Microsoft Excel for subsequent screening.

**Table 1:** Database formula during literature search

**PubMed Formula**
((“Post-COVID-19 syndrome“[Title/Abstract] OR “Post-COVID syndrome“[Title/Abstract] OR “Long COVID-19“[Title/Abstract] OR “Long COVID“[Title/Abstract] OR “persistent COVID-19“[Title/Abstract] OR “chronic COVID-19“[Title/Abstract] OR “Long COVID-19 symptoms“[Title/Abstract] OR “COVID-19 sequelae“[Title/Abstract])) AND ((“cardiovascular disease*“[Title/Abstract] OR “hypertension“[Title/Abstract] OR “heart disease*“[Title/Abstract] OR “angina“[Title/Abstract] OR “myocardial infarction“[Title/Abstract] OR “heart failure“[Title/Abstract] OR “heart attack“[Title/Abstract] OR “isch? emia“[Title/Abstract]))
**Scopus Formula**
( TITLE-ABS-KEY ( ( “Post-COVID-19 syndrome” OR “Post-COVID syndrome” OR “Long COVID-19” OR “Long COVID” OR “persistent COVID-19” OR “chronic COVID-19” OR “Long COVID-19 symptoms” OR “COVID-19 sequelae” ) ) AND TITLE-ABS-KEY ( ( “cardiovascular disease*” OR “hypertension” OR “heart disease*” OR “angina” OR “myocardial infarction” OR “heart failure” OR “heart attack” OR “isch? emia” ) ) ) AND PUBYEAR > 2019 AND PUBYEAR < 2024 AND ( LIMIT-TO ( DOCTYPE, “ar” ) )
**Web of Science Formula**
(“Post-COVID-19 syndrome” OR “Post-COVID syndrome” OR “Long COVID-19” OR “Long COVID” OR “persistent COVID-19” OR “chronic COVID-19” OR “Long COVID-19 symptoms” OR “COVID-19 sequelae”) (Topic) and (“cardiovascular disease*” OR “hypertension” OR “heart disease*” OR “angina” OR “myocardial infarction” OR “heart failure” OR “heart attack” OR “isch? emia”) (Topic) and Article (Document Types)

### Eligibility criteria

One author selected the studies independently following the PICO (population, interest, comparator, outcome) framework. A second author resolved any uncertainties. Only original articles with observational study designs were included. Hence, reviews, case reports, pre-prints, editorials, research letters, and other non-original publications were excluded. Additionally, articles were excluded if the full text was not available and if the publication was non-open access. The review was limited to human studies and articles written in English. Thus, only studies that included adult patients (≥ 18 years old) with confirmed COVID-19 infection by nasopharyngeal swab PCR testing and that reported the association of CVD with persisting COVID-19 symptoms after at least 12 weeks from the recovery of acute COVID-19 infection were included. Investigations involving children (< 18 years old) and studies with < 100 participants were excluded to prevent small study effects.

### Data screening and extraction

Cross-sectional, case-control, and observational cohort studies were included in this review. These studies investigated COVID-19 survivors in outpatient or inpatient settings and assessed whether there were risk factors for COVID-19 symptoms that persisted for > 12 weeks after the initial infection. After obtaining the articles from the database searches, two authors removed all duplicates and independently screened the article titles and abstracts. The full text of all relevant articles was retrieved and analysed. The reference lists of the included articles were manually screened using the eligibility criteria.

**Fig. 1:**
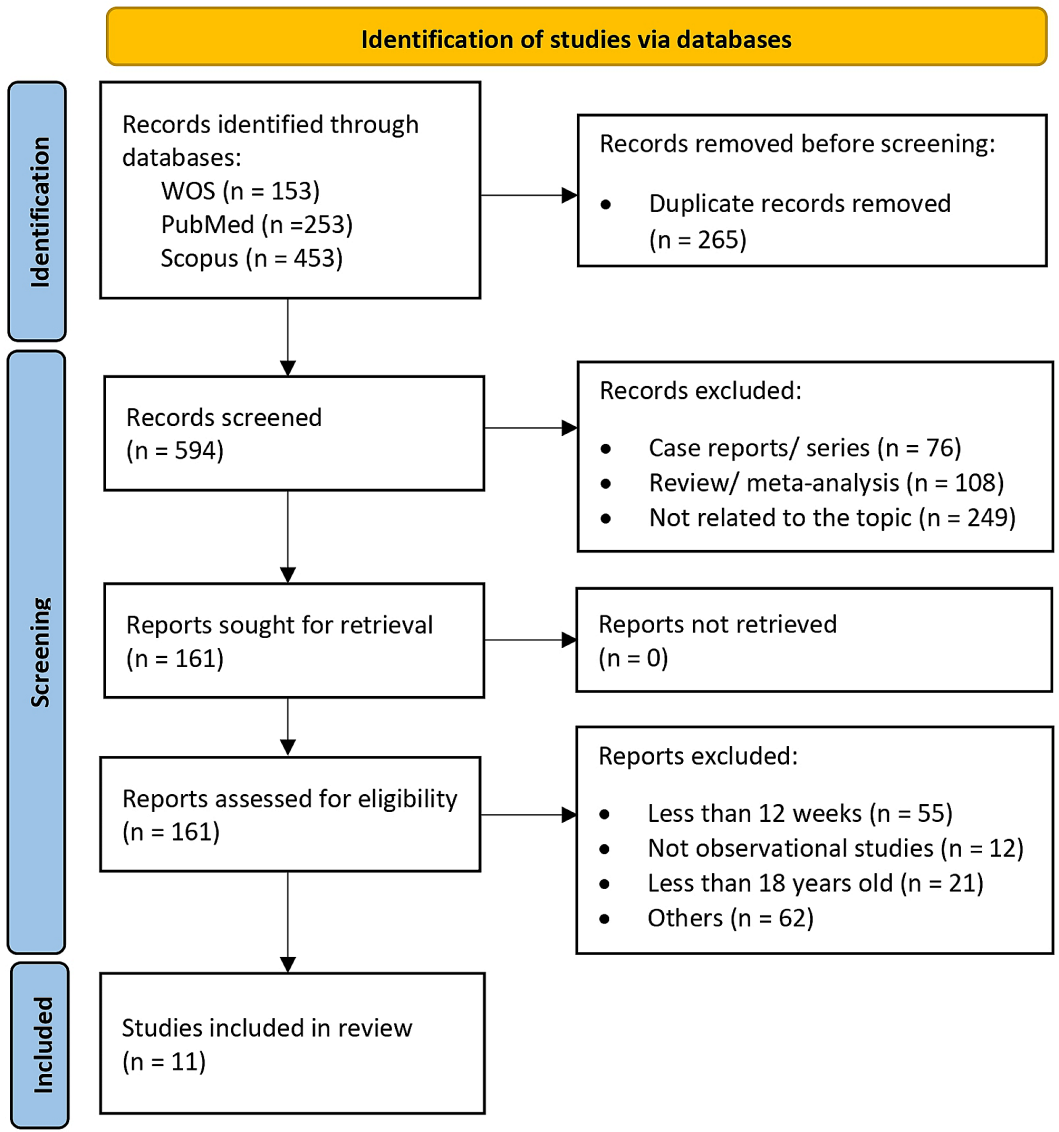
PRISMA flowchart of the study selection

The following data from each study was extracted to Microsoft Excel: (1) general information, (authors, country), (2) study characteristics (year, country, study design, sample size, mean or median age, percentage of female participants), (3) assessment of symptoms; (4) follow-up duration, and (5) outcomes of the risk factors for PCS. A third author was consulted and resolved any discrepancies between reviewers during the screening and data extraction. Figure 1 illustrates the PRISMA flowchart of the study selection process and depicts the total number of retrieved publications and the number of included and excluded studies.

### Methodological quality assessment

The methodological quality of the included studies was evaluated using the Newcastle-Ottawa Scale (NOS). Studies were scored on overall quality with 0 (minimum) to 8 (maximum) points according to NOS criteria. The studies were assessed critically based on each NOS domain (selection, comparability, outcome). Subsequently, the quality scores were ranked as poor (0–3), moderate [4–6], or high (7–8).

### Data synthesis and analysis

The review results are presented descriptively, and the odds ratio (OR) was analysed. We used a standardised mean difference and effect sizes and their 95% CI for continuous data. The eligible studies are compiled in tables that outline the overall study features and main findings to achieve thorough and transparent reporting and to facilitate interpretation. We included significant *p*-values from the multiple-regression model data, however, if the studies absent of this model, we used bivariate analysis data to identify the association between CVD and PCS.

## Results

### Study selection

A total of 859 references were retrieved from the electronic databases and uploaded to EndNote for automated duplication checks, where all duplicate articles and ineligible publication types (*n* = 265) were discarded. Subsequently, 594 references remained for the topic screening. Of these, 422 references were excluded: 76 case reports or case series, 108 reviews or meta-analysis studies, and 249 unrelated addressed topics. Next, the full text of the remaining 161 articles were reviewed, where 150 articles were removed, leaving 11 articles that were ultimately included in the review. Disagreements regarding the article’s inclusion or exclusion were resolved via internal discussions among the authors.

### Study characteristics

Table 2 summarises the 11 included studies, which were conducted in the US (three studies) and France, Brazil, Italy, China, Poland, India, Saudi Arabia, and the UK (one study each). The included studies were principally observational studies: seven prospective cohorts, three retrospective cohorts, and one cross-sectional, which examined the CVD factors associated with PCS. The studies had differing mean or median ages, sample sizes, proportions of female participants, symptom assessments, and follow-up durations. The included studies had sample sizes of 100–916,894 participants. The mean or median age was > 50 years (seven studies), < 50 years (two studies), and was not mentioned in the remaining two studies. The percentage of female participants was 10.9–60.8%.

**Table 2:** Main characteristics of the included studies

Authors	Year	Country^a^	Study design^b^	Sample size (*n*)	Age (mean/ median)	Sex (% female)	Symptoms assessment^c^	Follow-up duration	Main findings^d^
Ko et al. (2022) [22]	2020	France	PC	316	Mean age 64 years	46.3%	Medical record, clinical evaluation	4 months	Hypertension: OR = 1.64 (95% CI: 1.04–2.61; *p* = 0.04)
Freire et al. (2022) [23]	2020	Brazil	PC	822	Median age 56 years	47%	Medical record, clinical evaluation	6 months	Hospital readmission associated with: Congestive heart failure: OR = 2.34 (95% CI: 1.29–4.23; *p* = 0.005) Peripheral arterial disease: OR = 2.06 (95% CI: 0.97–4.38; *p* = 0.06)
Ioannou et al. (2022) [24]	2020	USA	RC	198,601	Mean age 60.4 (SD 17.7) years	10.9%	Medical record	8 months	Congestive heart failure: aOR = 1.34 (95% CI: 1.28–1.41) Myocardial infarction: aOR = 1.28 (95% CI: 1.18–1.38) Peripheral arterial disease: aOR = 1.23 (95% CI: 1.18–1.28) Venous thromboembolism: aOR = 1.30 (95% CI: 1.21–1.40)
Jakubowska et al. (2022) [25]	2020	Poland	PC	796	Median age 51 (41–62) years	60.8%	EMR, clinical evaluation	NA	History of myocardial infarction: OR = 2.57 (95% CI: 1.04–6.32)
Okoye et al. (2023) [26]	2020	Italy	PC	100	Mean age 85 (SD 4) years	42%	CGA, CIRS-c, HGS, SPPB	12 months	Chronic heart failure: aOR = 3.00 (95% CI: 1.10–8.16; *p* = 0.031)
Patel et al. (2022) [27]	2020	USA	PC	778,738	Mean age 61 (SD 15.4) years	11%	Medical record, CCI	3 months	**Hospitalized**: Venous thromboembolism: OR = 2.1 (95% CI: 1.8–2.4, *p* < 0.001) Cardiac arrythmia and hypertension: OR = 1.5 (95% CI: 1.5–1.6, *p* < 0.001) **Outpatient**: Venous thromboembolism: OR = 2.4 (95% CI: 2.1–2.7, *p* < 0.001) Cardiac arrhythmia: OR = 1.3 (95% CI: 1.2–1.4, *p* < 0.001) Ischaemic heart disease: OR = 0.8 (95% CI: 0.7–0.9, *p* < 0.001) Peripheral vascular disease: OR = 0.7 (95% CI: 0.6–0.8, *p* < 0.001)
Sedgley et al. (2023) [28]	2020	USA	RC	916,894	Mean age 47.9 (SD 21.4) years	59.6%	EMR, CCI	20 weeks	Hypertension: OR = 1.47 (95% CI: 1.44–1.49)
Shukla et al. (2023) [29]	2021	India	CS	1,223	Mean age 31.49 (SD 9.54) years	50.81%	EMR, DASS-21	13 months	Hypertension: OR = 2.06 (95% CI: 1.07–3.96, *p* = 0.029)
Tleyjeh et al. (2022) [30]	2020	Saudi Arabia	RC	222	NA	23.1%	MRC dyspnoea scale, MET score, CFS scale, WHO-5	18 weeks	Hypertension: OR = 0.40 (95% CI: 0.18–0.87)
Zhang et al. (2022) [31]	2021	China	PC	248	Median age 61 (54–68) years	54.8%	Medical record, clinical evaluation	14 weeks	Hypertension associated with: Fatigue: OR = 2.51 (95% CI: 1.08–5.80, *p* = 0.03) SOB: OR = 2.34 (95% CI: 1.16–4.69, *p* = 0.02) Palpitations: OR = 2.82 (95% CI: 1.26–6.31, *p* = 0.01) Expectoration: OR 2.08 (95% CI: 1.01–4.30, *p* = 0.04) Sore/dry throat: OR 2.71 (95% CI: 1.30–5.65, *p* = 0.01)
Zheng et al. (2023) [32]	2020	UK	PC	990	NA	44.7%	Medical record, MRC dyspnea scale	1 year	Cardiovascular disease: OR = 1.69 (95% CI: 1.27–2.25)

**Notes**:

^a^ Country, USA: United States of America; UK: United Kingdom

^b^ Study designs, CS: Cross-sectional; PC: Prospective cohort; RC: Retrospective cohort

^c^ CCI: Charlson comorbidity index; CFS: chronic fatigability syndrome; CGA: comprehensive geriatric assessment; CIRS-s: Cumulative Illness Rating Scale; EMR: Electronic medical records; HGS: handgrip strength test; MET: metabolic equivalent of task; MRC: Medical research council; SPPB: short physical performance battery; WHO-5: WHO-five well-being index

^d^ Main findings, SOB: Shortness of breath

### Main findings

The 11 articles, which involved > 1.5 million COVID-19 survivors, were analysed to determine the association between PCS and CVD (Table 2). The pre-existing CVD were hypertension (six studies), congestive or chronic heart failure (three studies), myocardial infarction, peripheral arterial disease, venous thromboembolism (two studies each), peripheral vascular disease, cardiac arrhythmia, ischaemic heart disease, and non-specific CVD (one study each). Six studies reported an association between a single CVD and the presence of PCS, while five studies reported multiple CVD and the presence of PCS. The data were presented as means, medians, percentages, and OR. All included studies presented the main findings with the OR.

### Single CVD and PCS

Six articles reported an association between a single CVD and PCS development. Ko et al. reported that hypertension was associated with more frequent persistent symptoms (OR 1.64; 95% CI 1.04–2.61; *p* = 0.04) [22]. Moreover, the greater likelihood of long COVID symptoms 12–20 weeks after the index acute COVID-19 infection was most strongly correlated with hypertension (OR 1.47; 95% CI 1.44–1.49). The PCS condition involves multi-system symptoms such as joint stiffness, cough, fatigue, and chest pain [3, 6–8, 28]. Additionally, hypertensive patients had higher odds of post-COVID sequelae compared to non-comorbid patients (OR 2.06; 95% CI 1.07–3.96, *p* = 0.029) [29].

Hypertension was also associated with lower metabolic equivalent of task (MET) exercise tolerance scores (OR 0.40; 95% CI 0.18–0.87) during follow-up. Ten variables that ranged from at rest to performing simple activities were assessed [30]. Hypertension was another potential risk factor for post-COVID sequelae 1 year after discharge. Hypertension was attributed to an increased risk of fatigue (OR 2.51; 95% CI 1.08–5.80, *p* = 0.03), shortness of breath (OR 2.34; 95% CI 1.16– 4.69, *p* = 0.02), palpitations (OR 2.82; 95% CI 1.26–6.31, *p* = 0.01), expectoration (OR 2.08; 95% CI 1.01–4.30, *p* = 0.04), and sore throat (OR 2.71; 95% CI 1.30–5.65, *p* = 0.01) [31]. Due to the necessary reduction in fundamental daily activities and a higher rate of complications than COVID-19 survivors without chronic heart failure, COVID-19 survivors with chronic heart failure required rehospitalisation. Therefore, COVID-19 patients with chronic heart failure had a three-fold greater rate of rehospitalisation compared to the controls [26]. Furthermore, myocardial infarction was substantially more frequently reported in patients with PCS (OR 2.57; 95% CI 1.04–6.32) [25].

### Multiple CVD and PCS

Freire et al. observed that patients with congestive heart failure and peripheral arterial disease commonly developed persistent myalgia, cough, and diarrhoea within the follow-up duration [23]. This was consistent with data that revealed that long COVID care (general care, mental health care, specialty treatment in the prior 2 years) was associated not only with congestive heart failure, but also with myocardial infarction, peripheral arterial disease, and venous thromboembolism [24].

Among hospitalised and outpatient COVID-19 patients, venous thromboembolism and cardiac arrhythmia were significantly associated with a higher incidence of either physical or mental health conditions as compared to that in COVID-19-negative patients. Similarly, hospitalised COVID-19 patients with hypertensive conditions and outpatient COVID-19 patients with ischaemic heart disease and peripheral venous disease had a significant likelihood of developing new physical or mental health conditions, respectively [27]. The physical health conditions included fatigue, dyspnea, chest pain, myalgia, and cough, meanwhile, mental health conditions such as insomnia, panic disorder, cognitive blunting, and depressive disorder [3, 5, 27]. Zheng et al. reported a higher Medical Research Council (MRC) dyspnoea score in patients with CVD, which was consistent with increased odds of worsening dyspnoea over 1 year in those with CVD (OR 1.69; 95% CI 1.27–2.25) [32].

### Methodological quality assessment

NOS assessment of the methodological quality of the included studies revealed that they were of moderate or high quality (Table 3), i.e. ≥6 points. The studies were ranked from low to high according to methodological quality to highlight the most reliable data.

**Table 3:** Methodological quality assessment of included studies

Authors	Study design^a^	Newcastle-Ottawa Scale			Quality score
		Selection	Comparability	Outcome	
Zhang et al. (2022) [31]	PC	3	1	2	6
Freire et al. (2022) [23]	PC	4	1	2	7
Patel et al. (2022) [27]	RC	3	1	3	7
Jakubowska et al. (2022) [25]	PC	3	1	3	7
Shukla et al. (2023) [29]	CS	3	1	3	7
Tleyjeh et al. (2022) [30]	RC	3	1	3	7
Zheng et al. (2023) [32]	PC	3	1	3	7
Ko et al. (2022) [22]	PC	4	1	3	8
Ioannou et al. (2022) [24]	RC	4	1	3	8
Okoye et al. (2023) [26]	PC	4	1	3	8
Sedgley et al. (2023) [28]	RC	4	1	3	8

**Notes**: ^a^ CS: Cross-sectional; PC: Prospective cohort; RC: Retrospective cohort

## Discussion

### Post-COVID syndrome

Following the COVID-19 outbreak, a significant proportion of COVID-19 survivors worldwide developed persistent symptoms for up to 1 year past the initial acute infection phase, a disorder known as PCS [7, 33]. This phenomenon indicates that managing COVID-19 aftereffects is crucial. COVID-19 sequelae may range from mild to severe persistent symptoms. Some patients might develop mild symptoms, commonly fatigue, cough, headache, muscle pain, cognitive problems, insomnia, and psychological symptoms 12 weeks from the initial COVID-19 infection [5, 34], while patients who develop chronic symptoms, including shortness of breath, chest pain, and diarrhoea, might require rehospitalisation. Over time, these symptoms might fluctuate, flare up, or relapse, negatively affecting multiple organ systems [34–36].

### CVD and PCS

Numerous studies have reported that even after the acute infection has been treated, many COVID-19 survivors with comorbidities might continue to have new or ongoing symptoms, which might be directly related to pre-infection factors. Arjun et al. reported that a higher incidence of PCS symptoms was correlated to various comorbidities [37]. The comorbidities with the highest association were hypertension, diabetes mellitus, cardiac problems, asthma, kidney problems, other pulmonary disorders, and cancer. Hence, most patients continue to experience productivity difficulties in their daily life > 12 weeks after acute infection [38].

The present review investigated the relationship between PCS and premorbid conditions, particularly CVD, in PCS patients globally. The results supported prior findings that CVD is associated with PCS development. The key results of the present review were that patients with pre-existing CVD were more likely to experience PCS. The prevalence of CVD highlighted the complex burden of PCS on individuals. Furthermore, we determined that all included studies reported disparate outcomes, which limited the therapeutic application of this knowledge. The evidence from this systematic review suggested that CVD such as hypertension, congestive or chronic heart failure, myocardial infarction, transient ischemic attack, peripheral arterial or venous disease, venous thromboembolism, cardiac arrhythmia, and ischaemic heart disease are associated with a higher likelihood of developing mild to severe PCS. Thus, PCS patients with these pre-existing CVD were identified as a high-risk group for acquiring PCS symptoms.

In this study, hypertension was associated with higher risks for most post-infection symptoms [23, 27–31]. In fact, hypertension was a significant risk factor for multiple sequelae such as fatigue, cough, palpitations, chest tightness, and shortness of breath [39, 40]. Consequently, COVID-19 survivors with hypertension might endure similar symptoms for an extended period [41]. Moreover, following an acute COVID-19 infection, patients with underlying cardiovascular issues may require longer recovery times. Research has demonstrated that those with CVD are more likely to experience serious conditions and may need longer hospital stays, both of which can prolong the duration of PCS [42, 43].

Additionally, common cardiac involvement arose due to fluctuating heart rates and blood pressure responses to clinical assessments. COVID-19 has been related to electrocardiographic abnormalities, which include right ventricular dysfunction (26.3%), left ventricular dysfunction (18.4%), diastolic dysfunction (13.2%), and pericardial perfusion (7.2%). These issues might impair heart function, elevate blood pressure, and aggravate hypertensive conditions. It is unknown how much of this is reversible in patients who progress to PCS [44, 45]. Furthermore, the length of PCS in this high-risk group can be prolonged by long-term consequences like myocardial damage, myocarditis, and arrhythmias [46, 47].

Our findings indicated that heart failure and peripheral arterial or venous disease resulted in hospital readmission or the requirement for PCS medical care due to severe persistent symptoms such as coughing and diarrhoea [23, 24, 26]. This was consistent with the outcomes of a previous study, which reported that a diagnosis of heart failure increased the likelihood of readmission within 60 days of being discharged by four times [48]. Similarly, chronic heart failure was the predominant clinical feature that caused rehospitalisation, with approximately 3-fold higher prevalence of rehospitalisation compared to the control group. Rey et al. also observed that SARS-CoV-2 potentially causes cardiac injury and could lead to increased recurrent acute decompensation, especially in older people with compromised baseline cardiopulmonary features [49]. Whether COVID-19 directly damages the myocardium or pre-existing heart failure explains this connection continues to be debated [50–52].

Our study also revealed that COVID-19 survivors with myocardial infarction had difficulty performing regular activities and had sleep problems, which affected their quality of life [24, 25]. The most prevalent abnormality detected with acute COVID-19 infection is myocardial damage, which is typically identified when patients have increased cardiac troponin levels and might be present in a significant percentage of COVID-19 patients [53, 54]. Furthermore, PCS patients might experience chest pain (17%), palpitations (20%), and dyspnoea with exertion [45, 55]. The incidence of cardiac arrhythmias in PCS is unknown, but some patients could experience palpitations [56]. Additionally, cardiac sequelae from acute COVID-19, such as peripheral arterial or venous disorders, coronary artery aneurysms, and arterial or venous thromboembolism, can emerge in PCS patients long after recovery from acute illness. These anatomical anomalies can cause shortness of breath and chest pain or tightness [44]. Symptom frequency might decrease as the infection progresses, which renders the timing of the assessment crucial [4].

Besides, our data emphasised that patients with hypertension, heart failure, heart attack etc. have illuminated the severity of PCS and the urgency of this problem. Policymakers can comprehend the full extent of the problem and its effects on public health by measuring the burden of this issue. Furthermore, our findings also can help design holistic public health strategies that resolve several factors influencing health, such as medication accessibility, rehabilitative medical treatment, and at-home physical activity [57, 58]. Long-term monitoring and management strategies are crucial for those with pre-existing CVD, given the possible impact of CVD on the duration and course of PCS. Several guidelines have been established by different organisations in various countries for managing high-risk groups that require close monitoring of cardiac function, risk factor prevention, and multidisciplinary care coordination [59, 60].

### Strengths, limitations and future directions

A strength of our study is that we comprehensively searched all currently available evidence with a focus on the correlation between multiple pre-existing CVD and a higher risk of acquiring PCS, which resulted in the inclusion of 11 studies in this review. Our main findings demonstrated the characterisation of PCS symptomatology in different populations and highlighted its implications concerning various CVD types. It underscores how crucial it is to strengthen the comprehensive approach to emphasise that several disciplines must assist PCS patients. This is a helpful way to demonstrate our dedication to providing patients with holistic care. In addition, the methodological quality evaluation of the included studies was rated as good quality and was deemed to be mostly satisfactory.

A major limitation of this review was that the included studies had substantially heterogeneous study designs, demographics, settings, mean ages, sample sizes, symptom testing techniques, and follow-up intervals. Furthermore, the combination of cohort sampling and data-collecting procedures could have yielded inconsistent and diverse results. However, our results were consistent and reflected the association between CVD and PCS. Additionally, we identified studies that primarily used validated definitions of PCS for standardisation, which was similar to Greenhalgh et al. [7]. These findings emphasised the need for more research with improved confounding factor control, coordinated PCS evaluation tools, and the use of a consistent and validated definition of PCS to improve quality standards and minimise reporting heterogeneity.

Future research needs to homogenise methods and data collection to ensure the outcome is more reliable and relevant. Since our findings are based primarily on observation studies, which resulted in a small number of included studies, we advise future research to incorporate other study designs to gain more reliable information and diverse points of view in different populations. Our work underscores the necessity of more research involving a wider range of populations. Subsequent investigations ought to concentrate on clarifying the processes that underlie the reported impacts of PCS. Future research may change clinical practice standards and enhance patient care by pursuing these research goals and advancing the field’s understanding.

## Conclusion

Our systematic review indicated that CVD might be the risk factor for the emergence of PCS. Currently, COVID-19 survivors with pre-existing CVD such as hypertension and heart failure have a greater likelihood of developing PCS than COVID-19 survivors without CVD. PCS clearly affects various populations and exhibits a broad spectrum of symptoms. We wish to highlight the fact that the burden of PCS will escalate as more people with CVD develop the condition. Additionally, as heart failure, hypertension, and other CVD are associated with a higher risk of developing PCS, it is necessary to screen and examine these patients more thoroughly and early on during follow-up sessions to improve their outcomes. These groups at risk also need comprehensive healthcare management and effective preventative strategies to reduce the likelihood of any inaccurate measures. Given the challenging circumstances and negative effects of PCS on an individual, additional research is suggested to obtain more knowledge and create specific guidelines for certain populations, such as those with premorbid conditions.

### Electronic supplementary material

Below is the link to the electronic supplementary material.

Supplementary Material 1

## Data Availability

All data relevant to the study are included in the article or uploaded as online supplementary information. The protocol of this systematic review can be found at https://www.crd.york.ac.uk/prospero/display_record.php?ID=CRD42023440834.

## Abbreviations

CVD: Cardiovascular diseases
CCI: Charlson Comorbidity Index CFS: Chronic fatigability syndrome
CGA: Comprehensive geriatric assessment
CI: Confidence interval
COVID-19: Coronavirus disease 2019
CS: Cross-sectional
CIRS-s: Cumulative Illness Rating Scale
EMR: Electronic medical records
HGS: Handgrip strength test
HR: Hazard ratio
MRC: Medical Research Council
MeSH: Medical subject heading
MET: Metabolic equivalent of task
NOS: Newcastle-Ottawa Scale
PICO: Population, interest, comparator, outcome
PCS: Post-COVID syndrome
PRISMA: Preferred Reporting Items for Systematic Review and Meta-Analyses
PC: Prospective cohort
RC: Retrospective cohort
SARS-CoV-2: severe acute respiratory syndrome coronavirus 2
SPPB: Short physical performance battery
SOB: Shortness of breath
UK: United Kingdom
USA: United States of America
WOS: Web of Science
WHO-5: WHO-five well-being index
